# Supplementary material for: Gap junction plasticity as a mechanism to regulate network-wide oscillations
Source: PLoS Comput Biol. 2018 Mar 12;14(3):e1006025. doi: 10.1371/journal.pcbi.1006025 (PMC5864095; doi:10.1371/journal.pcbi.1006025)
Supplement: S1 Text — Additional methods in relation to the supporting information. (PDF) [file pcbi.1006025.s002.pdf]

Gap junction plasticity as a mechanism to regulate network-wide oscillations

Guillaume Pernelle[1], Wilten Nicola[1], Claudia Clopath[1,*],

1 Bioengineering Department, Imperial College London, London, United Kingdom

* c.clopath@imperial.ac.uk

Supporting information

Passive rules as gap junction long term potentiation.

We consider two additional gap junction long term potentiation (gLTP) rules, which are passive as opposed to activity-dependent in the main article. In the passive case, the gap junction coupling increases constantly by a small amount. We consider a soft bound, i.e. the magnitude of increase is proportional to the difference between the gap junction value and a baseline coupling strength γ_b.

$$\dot{\gamma}^+_{ij}(t) = \dot{\gamma}^+_{ji}(t) = \alpha_{LTP} \left(\frac{\gamma_b - \gamma_{ij}(t)}{\gamma_b} \right). \tag{1}$$

Second, we consider a gLTP rule with no bounds, written as

$$\dot{\gamma}^+_{ij}(t) = \dot{\gamma}^+_{ji}(t) = \alpha_{LTP}. \tag{2}$$

Asymmetrical gap junction plasticity.

We consider plasticity rules where the change is directional, and not symmetrical as previously described. After the bursting protocol, (*Haas et al. 2011*) (Fig. 4C) measured a larger coupling change for the outbound coupling, ie. when the coupling is measured with current injection in the cell that was quiet during the pairing protocol. In light of this observation, we consider the following gap junction long term depression (gLTD) rule

$$\dot{\gamma}^-_{ij}(t) = -\alpha^*_{LTD}[H(b_j(t) - \theta_{burst})], \tag{3}$$

where α^*_{gLTD} is the learning rate, H is the Heavyside that returns 1 for positive arguments, b_j is the same low-pass filter of the neuron activity as described in equation (12) in the main text and θ_{burst} is a bursting threshold. To compare this rule with the symmetrical rule, we set $\alpha^*_{gLTD} = 2 \cdot \alpha_{LTD}$, as overall there is twice less activity driving plasticity. As for the main text, the parameters are fit by implementing the stimulation protocol used by Haas et al.

The activity-dependent gLTP rules becomes

$$\dot{\gamma}^+_{ij}(t) = \alpha_{LTP} \left(\frac{\gamma_b - \gamma_{ij}(t)}{\gamma_b} \right) [\mathrm{sp}_j(t)], \tag{4}$$

where α_{gLTP} is the learning rate and $\mathrm{sp}_i(t) = \sum_{t_{ik}<t} \delta(t - t_{ik})$ and δ is the Dirac function. The unbounded gLTP rule becomes

$$\dot{\gamma}^+_{ij}(t) = \alpha_{LTP}[\mathrm{sp}_j(t)]. \tag{5}$$

Additional parameters.

We list in Table S1 the parameters used for our simulations in the supplementary material. Other parameters, except those described in the figure captions, remained unchanged. The symmetrical and asymmetrical rules have the same parameters, except for α_{gLTD} becoming α^*_{gLTD} for asymmetrical gap junctions. As reported in the main text, $\alpha_{gLTD} = 15.69$ nS $\cdot$ ms^{-1}.
