# Supplementary figures and images for: Gap junction plasticity as a mechanism to regulate network-wide oscillations
Source: PLoS Comput Biol. 2018 Mar 12;14(3):e1006025. doi: 10.1371/journal.pcbi.1006025 (PMC5864095; doi:10.1371/journal.pcbi.1006025)

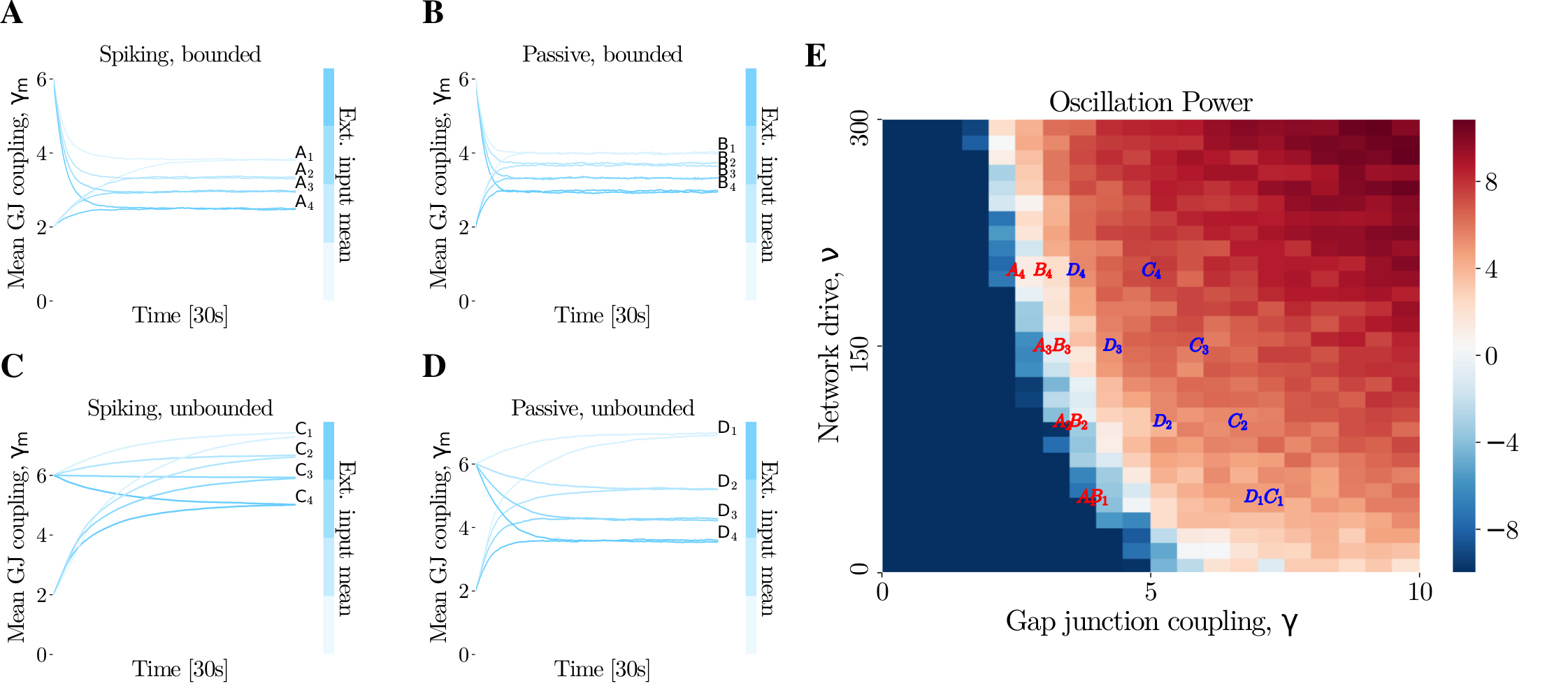

Supplement: S1 Fig — Four gLTP rules are considered: potentiation is either soft-bounded (A, B) or unbounded (C, D) and the rule is activity-dependent (A,C) or passive (B, D). The evolution of the mean gap junction coupling is represented over time, for different initial values (γm(0) = {2, 6}) and mean network drive ν. The network drive is a colored noise current Inoise described in Eq (11) in the main text. The value taken by ν are 50, 100, 150 or 200 pA. Lighter colors represent smaller values of ν. We observe that the value of the steady-state do not depend on the initial value of the mean gap junction coupling. (E) Power of the main frequency component in the Fourier domain of the population activity of inhibitory neurons for 2 seconds of simulation. The blue area denotes the lack of oscillations. Labels show the steady-state of the simulations designed by the same labels. Note that only the steady-states of simulations initialized with γm(0) = 2 are shown. (TIF) [file pcbi.1006025.s003.tif]

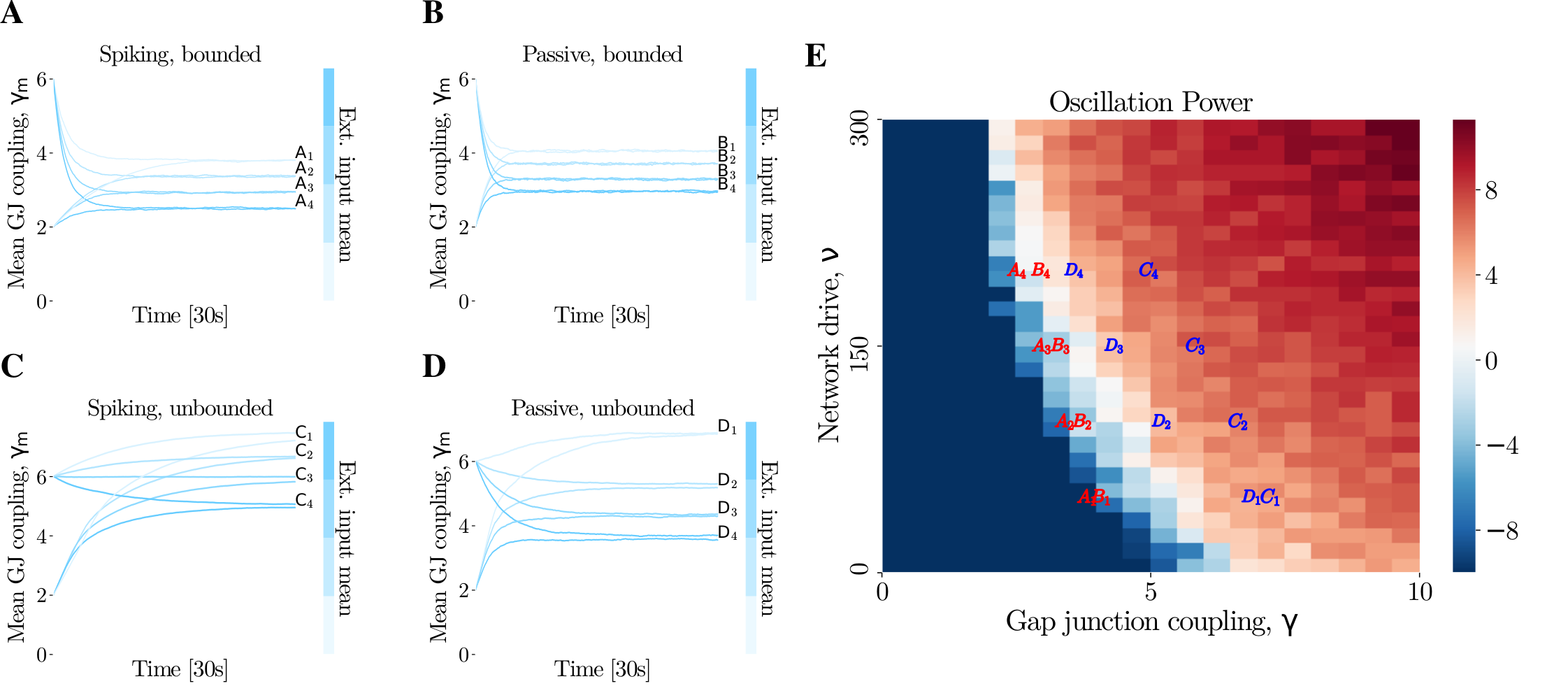

Supplement: S2 Fig — Same caption as S1 Fig, but for asymmetrical gap junction plasticity. Four gLTP rules are considered: potentiation is either soft-bounded (A, B) or unbounded (C, D) and the rule is activity-dependent (A,C) or passive (B, D). The evolution of the mean gap junction coupling is represented over time, for different initial values (γm(0) = {2, 6}) and mean network drive ν. The network drive is a colored noise current Inoise described in Eq (11) in the main text. The value taken by ν are 50, 100, 150 or 200 pA. Lighter colors represent smaller values of ν. We observe that the value of the steady-state do not depend on the initial value of the mean gap junction coupling. (E) Power of the main frequency component in the Fourier domain of the population activity of inhibitory neurons for 2 seconds of simulation. The blue area denotes the lack of oscillations. Labels show the steady-state of the simulations designed by the same labels. Note that only the steady-states of simulations initialized with γm(0) = 2 are shown. (TIF) [file pcbi.1006025.s004.tif]

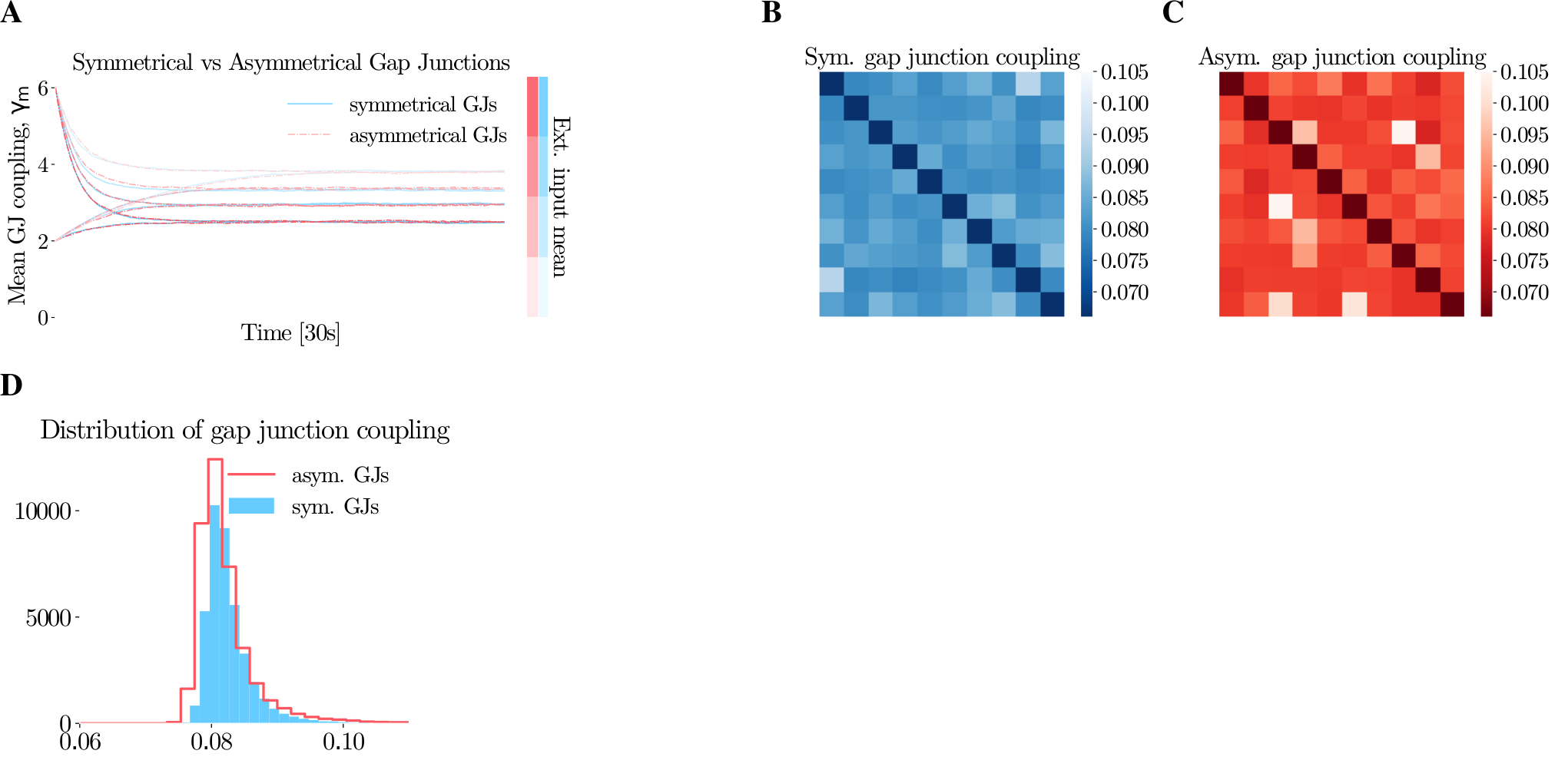

Supplement: S3 Fig — (A) Evolution of the mean gap junction strength when gap junctions are symmetrical (blue continuous lines, same simulations as for panel A of S1 Fig) or asymmetrical (red dashed lines, same simulations as for panel A of S2 Fig). Lighter colors represent lower values of the mean network drive, going from 50 pA to 200 pA. The results shown are for the activity-dependent, soft-bounded gLTP rule. The results are identical for the passive gLTP rules. The initial conditions (network drive and initial mean coupling) are the same as described for S1 and S2 Figs. (B,C) Weight matrix for 10 gap junctions once the mean gap junction coupling has reach its steady state, for symmetrical gap junctions (B) and asymmetrical gap junctions (C). Lights colors represent stronger values of the gap junction coupling. (D) Histogram of the individual gap junction coupling at steady state, for symmetrical gap junctions (blue), and asymmetrical gap junctions (red). (TIF) [file pcbi.1006025.s005.tif]

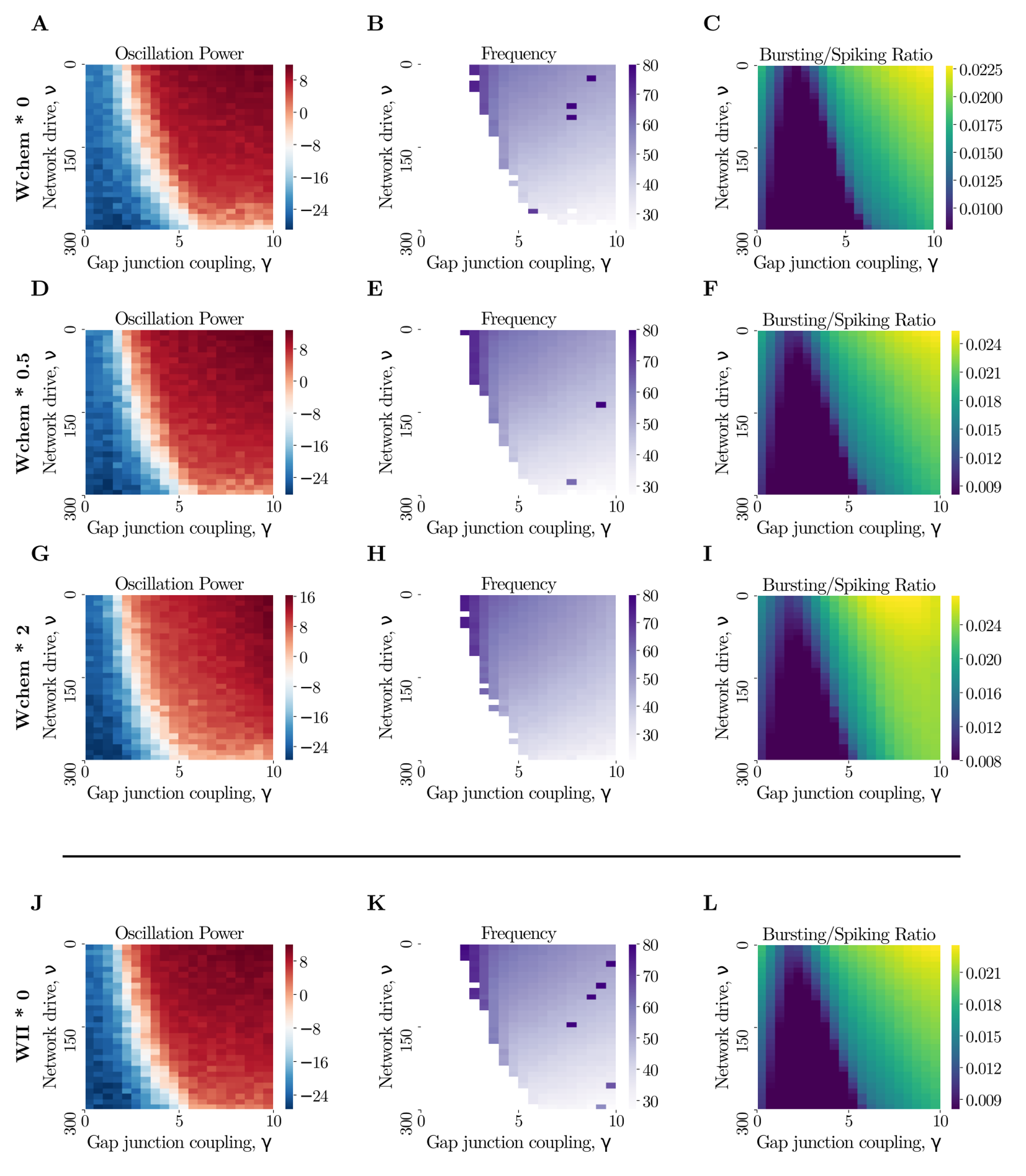

Supplement: S4 Fig — Phase diagrams as function of the chemical coupling: First column (A,D,G,J): Power of the main frequency component in the Fourier domain of the population activity (PA) of inhibitory neurons. Second column (B,E,H,K): Oscillation frequency of the network activity. The white area represents a region where the network is not oscillating and has no oscillation frequency. Third column (C,F,I,L): Ratio of bursting Abursting over spiking Aspiking activity, averaged over 2 seconds. Bursting activity prevails in the light region and sparse firing dominates in the dark region. First row (A,B,C): There is no chemical synapses. Second row (D,E,F): The strength of chemical synapses is half of the weights used for this study. Third row (G,H,I) The strength of chemical synapses is doubled. Last row (J,K,L): There is no inhibitory to inhibitory chemical synapses, but the rest of the chemical synapses have the standard strength. (TIF) [file pcbi.1006025.s006.tif]

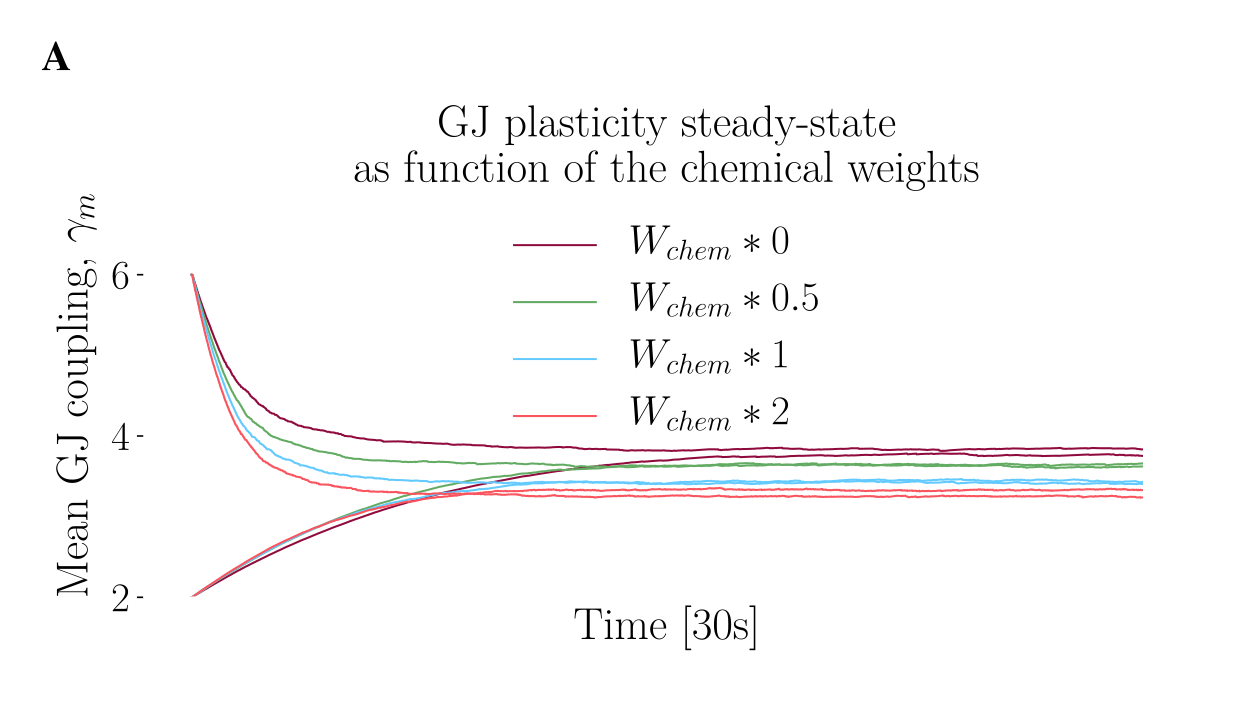

Supplement: S5 Fig — (A) Evolution of the mean gap junction strength during 30 seconds, when there is no chemical synapses (purple lines), or when the strength of the chemical synapses is halved (green lines) or doubled (red lines) in reference to the value used for this study (blue lines). The convergence of the plasticity steady-state is observed for 2 different values of the initial mean gap junction conductance which correspond to a network initialised in the synchronous or in the asynchronous regime. (TIF) [file pcbi.1006025.s007.tif]
